# Single-cell Landscape of Malignant Transition: Unraveling Cancer Cell-of-Origin and Heterogeneous Tissue Microenvironment

**DOI:** 10.21203/rs.3.rs-4085185/v1

**Published:** 2024-04-05

**Authors:** Ruihan Luo, Jiajia Liu, Jianguo Wen, Xiaobo Zhou

**Affiliations:** The University of Texas Health Science Center at Houston; School of Biomedical Informatics, The University of Texas Health Science Center at Houston; School of Biomedical Informatics, The University of Texas Health Science Center at Houston

**Keywords:** Precancer-to-Cancer Transition, Stem-like cells, Heterogeneity, Microenvironment, Macrophages, Fifibroblasts

## Abstract

Understanding disease progression and sophisticated tumor ecosystems is imperative for investigating tumorigenesis mechanisms and developing novel prevention strategies. Here, we dissected heterogeneous microenvironments during malignant transitions by leveraging data from 1396 samples spanning 13 major tissues. Within transitional stem-like subpopulations highly enriched in precancers and cancers, we identified 30 recurring cellular states strongly linked to malignancy, including hypoxia and epithelial senescence, revealing a high degree of plasticity in epithelial stem cells. By characterizing dynamics in stem-cell crosstalk with the microenvironment along the pseudotime axis, we found differential roles of ANXA1 at different stages of tumor development. In precancerous stages, reduced ANXA1 levels promoted monocyte differentiation toward M1 macrophages and inflammatory responses, whereas during malignant progression, upregulated ANXA1 fostered M2 macrophage polarization and cancer-associated fibroblast transformation by increasing TGF-β production. Our spatiotemporal analysis further provided insights into mechanisms responsible for immunosuppression and a potential target to control evolution of precancer and mitigate the risk for cancer development.

Carcinogenesis, the process by which normal cells evolve into malignant ones, involves the expansion of specific clones within both normal and diseased niches. This transformation is orchestrated by intricate interactions among immune, stromal, and precancer cells. Preventing cancer is the ultimate goal, necessitating a profound comprehension of precancer biology and a meticulous dissection of the disease development course. While large-scale genomics efforts have provided tremendous insight into fundamental neoplastic processes by documenting the commonality and diversity of various genetic and transcriptional alterations across diverse cancer types^1^, premalignant lesions have not been explored in depth. Currently, many breakthroughs have been made in precision prevention and therapeutics. For example, RANK-L inhibitors have shown promise in preventing/delaying mammary tumor onset^2^, and preventive cancer vaccines such as bivalent, quadrivalent, and nonavalent human papillomavirus (HPV) vaccines have proven effective in preventing high-grade cervical intraepithelial lesions (HSIL)^3–5^. These advances are attributed to the in-depth studies of the sequential molecular events and mechanisms underlying oncogenesis in BRCA1/2 + carriers and cervical intraepithelial neoplasia patients in the precancerous stage. Some interventions for specific cancer prevention have been approved by FDA, but these are not comprehensive enough to address various types of cancers. Moreover, researchers have made some progress in understanding cancer development and metastasis. However, existing databases, such as TISCH, predominantly focus on profiling invasive/advanced tumors, leaving a great deal of unknown about cancer predisposition and the complex interactions within the architecture of premalignant environment (PME). Therefore, in this study, we developed a core precancer-to-cancer transition atlas (PCTanno) by compiling 62 single-cell RNA sequencing (scRNA-seq) datasets comprising 1396 samples and covering 13 major tissue types. Global and tissue-/cell-type-specific biomarkers are invaluable in detecting early-stage lesions and dissecting the sequential molecular and cellular events that promote oncogenesis, paving the way for novel prevention and interception strategies. PCTanno provided comprehensive characterizations for genes involved in multistage tumorigenesis across diverse human tissue types and mapped cell composition and cell state changes during the malignant transformation from healthy tissues to precancer (e.g., cirrhosis) to cancer (e.g., HCC).

Accumulative evidence suggests that precancer stem cells arise from clonally mutated tissue stem cells that disrupt normal tissue homeostasis^6^. Aging, inflammation, and environmental stressors may induce precancer cells to undergo malignant reprogramming and acquire the so-called ‘self-renewal’ capacity^6^. This process may induce further phenotypic alterations in tissue stem cells, leading them to transition into cancer stem cells (CSCs). Hence, it becomes imperative to address the issues of when and how aging and systemic inflammation-related mechanisms govern the generation of precancer stem cells and their subsequent malignant transformation. In this context, we identified distinct stem-like cell subpopulations in diseased tissues that displayed appreciable variations in biophysical properties, such as subclusters of proliferating, EMT-like, and senescent cells during the malignant progression. Once oncogenic pathways are initiated, tissue stem cells can engage in abnormal molecular crosstalk with their surroufindings, culminating in a considerable remodeling of the tumor microenvironment (TME) during the benign-to-malignant transition^37^. Our analysis revealed that ANXA1 expression levels in stem cells were decreased from the healthy to the precancerous stages but gradually elevated along malignant transformation paths. This indicates different roles of ANXA1 in premalignant stages, where reduced ANXA1 levels foster proinflammatory cytokine production, leading to aggressive and/or prolonged inflammatory responses. In contrast, in carcinomas, it is upregulated in CSCs to promote the transformation of M1 macrophages into the M2 phenotype and normal fibroblasts into cancer-associated fibroblasts (CAFs) by increasing TGF-β production, potentially driving malignancy via FPR activation. Our spatiotemporal analysis delineated the forces driving tumorigenesis and provided insights into the tissue microenvironment for both precancer and cancer, including mechanisms of ‘homeostasis-imbalance-malignancy’ change during disease escalation.

## Results

### 1. Global view of single-cell transcriptome analyses of tumorigenesis in 13 tissues

**Figure 1:**
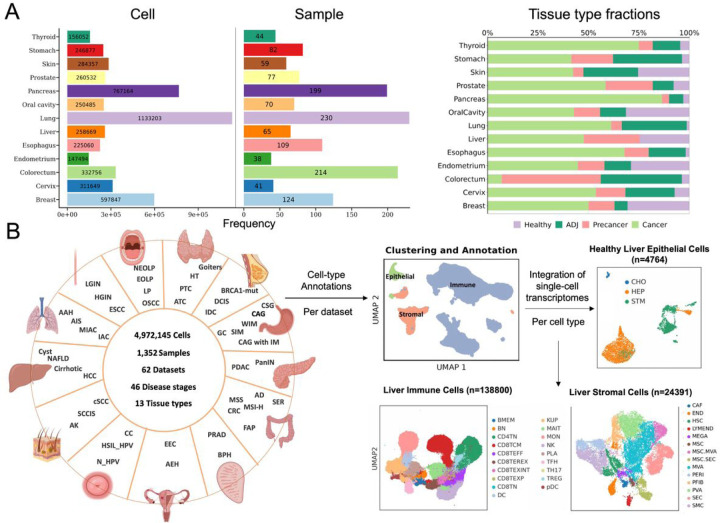
Construction of a multistage tumorigenesis single-cell RNA atlas. (A) Bar graphs showing summary statistics for the number of cells, samples collected by tissue (left) and their tissue compositions (right). (B) The schematic depicts the study design, data collection (left) and workflow of integration & annotation for scRNA-seq datasets (right, liver tissue as an example).

To systematically decipher the multistage tumorigenesis across various tissue types, we searched for and prioritized all publicly available studies that reported scRNA-seq data for human premalignant lesions, which covered 13 commonly affected tissues. We next collected transcriptomic datasets for corresponding healthy and cancerous samples. In total, these data encompassed 1,281 samples from 1,256 patients across adjacent non-tumor (ADJ), precancer and tumor, as well as 135 healthy controls from 57 studies. Following the removal of low-quality cells, we retained a total of 4,972,145 cells across different stages, integrating data to create a comprehensive resource (Fig. 1A). Subsequently, through sub-clustering cells from respective tissue types, stromal and immune cells were projected into low-dimensional subspaces and annotated with 71 major subtypes of tissue microenvironment cells. We analyzed healthy and diseased epithelial cells separately and annotated them into 58 tissue-specific cell types (see Method details). The annotation was based on previously established canonical marker genes (Table S1) following a pipeline from Granja et al. 2019^7^ and Becker et al. 2022^8^. To facilitate broader access and understanding, we established the PCTanno platform and provided all data at the website (https://ccsm.uth.edu/PCTfuncDB/index.html). PCTanno provides systematic functional annotations of the dynamic biological processes involved in tumorigenesis, including the cell annotations, cell composition by disease state, uniform manifold approximation and projection (UMAP) plots (Fig. 1B), inferred copy number variation (CNV), somatic mutations, and further downstream analyses described below. Our commitment is to consistently expand PCTanno by incorporating new datasets and functionalities across a diverse set of tissue types, to achieve a high-resolution understanding of clinically relevant transitions and gain new aspects of the development and evolution of malignancy.

### 2. Highly enriched stem-like cells in precancer and cancer stages

**Figure 2:**
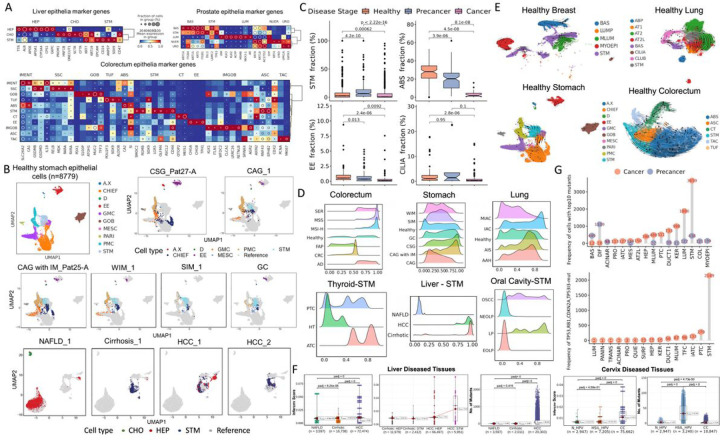
The meticulous dissection of epithelial compartment across diverse tissues along disease progression. (A) Heatmap of expression for representative marker genes of liver, prostate, and colorectum epithelia. (B) UMAP projection of scRNA-seq epithelial cells isolated from different disease stages of stomach tissues (CSG, CAG, CAG with IM, WIM, SIM, and GC) and liver tissues (NAFLD, cirrhosis, and HCC) into the manifold of healthy epithelial cells. Projected cells are colored by the nearest normal cells in the projection, and normal epithelial cells (reference) are colored gray. A.X, A/X cell; CHIEF, Chief cell; D, D cell; EE, enteroendocrine; GMC, Gland mucous cell; GOB, Goblet; MESC, metaplasia stem cell; PARI, parietal cell; PMC, Pit mucous cell; STM, stem-like cells; CHO, cholangiocytes; HEP, hepatocytes. (C) The proportions of four representative cell types in the epithelial lineage across stage groups. (D) Ridge plot of CytoTRACE score distributions for different stages of bulk samples (top) and for only stem-like cells (bottom). (E) UMAP showing the inferred development dynamics of healthy cells of 4 representative tissues by RNA velocity. (F) Comparisons of inferred CNV scores and mutation load among different stages and cell types of liver and cervix tissues. (G) Top, the frequency of the top 10 somatic mutations across different epithelial cell types detected in scRNA-seq data; bottom, the frequency of mutations from four representative cell cycle genes across cell types.

We analyzed epithelial cells from healthy donors and those with different disease states. The normal references composed of healthy epithelial cells from 13 tissues were constructed respectively, in which cell types were annotated using previously reported biomarkers. For example, there are stem cell populations with high expression of EPCAM, CD24, SOX9, ANPEP, and CD47 in the liver, DLL1, LAMC2, and TP63 in the prostate, LGR5, OLFM4, and ASCL2 in colorectum tissues (Fig. 2A). Then, the diseased cells were projected into the normal subspace. Intriguingly, epithelial cells from more malignant stages were prone to project closer to stem cells, whereas benign samples projected relatively evenly throughout the epithelial compartment (Fig. 2B). Moreover, many mature cell types (e.g., ABS in CRC, EE in GC and CRC, CILIA in EEC and IAC) were depleted from the cancer samples (Fig. 2C). This suggested an increasing stem-like phenotype existed in epithelial cells as they transformed from normal to more advanced lesions.

We employed CytoTRACE to predict differentiation states for each epithelial cell within samples. Healthy stem cells exhibited high CytoTRACE scores, transitioning into differentiated cells with lower scores (Fig. 2D, E, Figure S1A) and forming a relatively uniform distribution across stem-like, transitioning, and differentiated cells. In contrast, precancers (AD, CAG with IM, AAH) and cancers (MSS, GC, AIS and IAC) yielded a distribution skewed toward cells with high predicted stem potential (Fig. 2D). Stem-like cells from benign tissues such as NAFLD, NEOLP and N_HPV generally displayed lower stemness scores when compared with cirrhosis, LP, HSIL_HPV and tumors (indicating less differentiation) (Fig. 2D, Figure S1B). Interestingly, stem-like cells in precancers such as BPH, cirrhosis, and AK exhibited scores similar to those of cancers, even surpassing stemness scores in malignancies (e.g., BRCA1-mut and LP, Figure S1B). This similarity in stemness suggests the presence of CSCs. Remarkably, single cells from tumor samples exhibited a strikingly elevated CNV score, particularly conspicuous in hepatocarcinoma stem cells, accompanied by a higher mutation load compared to non-malignant cells within the epithelial compartment across most tissues (Fig. 2F, Figure S1B). This observation underscores the presence of significant aneuploid genomes and accumulation of mutations in the malignant stage. Conversely, abundant mutants were detected in premalignant lesions of the cervix, breast, and oral cavity (Fig. 2F, Figure S1C), indicating that persistent exposure to the mutagens, such as HPV virus and excessive hormones, contributes to chronic inflammation, inducing the DNA damage and genomic instability during the early tissue response to injury. The inference drawn is that HPV DNA integration into the host genome may confer a selective advantage to HPV-infected epithelial cells in this process. Certain mutations may endow a growth advantage or resistance to cell death, facilitating their persistence in the precancerous stage.

Shifting our attention to specific mutated genes associated with intratumor heterogeneity (ITH) across a thousand tumors^9^, we observed a noteworthy pattern. The top 10 mutated genes, including NCOR1 and NPM1 were frequently identified in stem-like cells, suggesting that stem- cell expansion often initiates tumorigenesis driven by these early mutations (Fig. 2G, Table S2). Furthermore, we noted that alterations in TP53, RB1, TP53I3 and CDKN2A, known to be associated with apoptotic responses and cell cycle arrest, were highly enriched in stem-like cells of tumors, were highly enriched in stem-like cells of tumors, such as in cases of ATC, DCIS and PDAC (Fig. 2G, Table S2). These findings propose that precancer cells within the tissuespecific stemcell compartment may give rise to an expanded progenitor population undergoing malignant regeneration and immune evasion, promoting the propagation of malignant stem cells in the context of extrinsic and intrinsic factors^6^. Nonetheless, how and when the cellular origins of tumors affect the development of precancerous lesions and clinical prognosis remain to be uncovered.

### 3. Mapping microenvironment cell composition and molecular changes along malignant transformation

**Figure 3:**
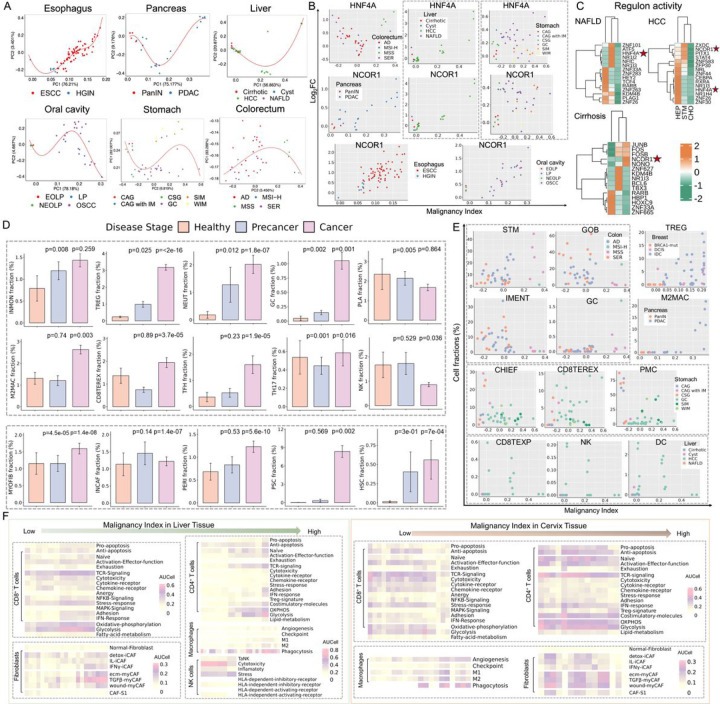
Identification and characterization of micro-environment cell composition and molecular changes along malignancy progression. (A)Trajectories for malignant transformation in scRNA-seq datasets of four representative tissues. (B) log_2_FC in expression of HNF4A and GPX2 in stem-like cells from each diseased sample relative to stem-like cells in healthy samples plotted against the malignancy continuum defined in A. (C) Heatmap showing the significantly different activities of TFs among different cell types in the liver-diseased tissues. Color overlays are regulon enrichment scores. (D) Comparisons of cell proportions of immune cells (top panel) and stromal cells (bottom panel) across stage groups in 13 tissues. (E) Fraction of cell types in each sample from five representative tissues with different disease stages plotted against the position of the sample in the malignancy index defined in A. (F) Heatmap illustrating expression changes of curated gene signatures across five subclusters along malignant transitions of the liver (left) and cervix (right) tissues.

Stem cells are recognized as pivotal contributors to cellular biological functions, possessing remarkable capacities for self-renewal and differentiation. However, dysregulation of self-renewal during aging and persistent microenvironmental and macroenvironmental stressors can lead to cancer^6^. This prompts an exploration into whether stem-like cells within the epithelial compartment can exhibit a continuum across diverse tissues and how the states of other cells in the tissue microenvironment dynamically change along this malignant continuum. To address this, we established a transcriptional malignancy index for each tissue type by identifying the differentially expressed genes (DEGs) in precancerous and cancerous lesions. Specifically, we compared the gene expression profiles of stem-like cells in diseased tissues with those in healthy donors. The obtained DEGs between the stem-like cells of each specimen and their healthy controls allowed us to calculate the principal components of the log_2_FC of these DEGs. Subsequently, we ordered the samples based on their positions along a spline fit in this space (Fig. 3A). The position in this ordering can be interpreted as the location/pseudo-time along a continuum ranging from healthy tissues to malignancies. Our analysis revealed a stereotyped progression in gene expression variations between normal and diseased stem-like cells, culminating in invasive tumors. Based on the expression dynamics analysis, we observed that the expression of HNF4A gradually increases as precancerous lesions approach malignant transformation in the gastrointestinal tract (Fig. 3B). Notably, different patterns of HNF4A usage were observed in precancer stages (e.g., AD v.s. SER, liver cirrhosis v.s. NAFLD, and SIM v.s. CAG). In precancers, HNF4A decreases to drive WNT signaling, while in carcinomas, it is upregulated in CSCs to drive malignancy. This aligns with previous findings associating HNF4A knockdown with an upregulation of WNT pathway signaling molecules, and HNF4A loss drives metabolic reprogramming at an early stage of pancreatic cancer progression^10^. Moreover, our SCENIC analysis^11^ identified HNF4A as a transcription factor (TF) specifically enriched in hepatocytes of HCC and NAFLD but not in healthy tissues, with its target genes (e.g., IRS1 and FGFR3) exhibiting similar expression dynamics (Fig. 3C, Figure S2A, Table S3). Consistent with prior work, increased gene expression and chromatin accessibility of HNF4A motifs were reported only after the transformation to CRC^8^. The forced re-expression of HNF4α, along with the application of all-trans retinoic acid, demonstrated effectiveness in reducing the number of liver CSCs and potentiating the chemotherapeutic effect^12^. As HNF4A acts as a selective agonist of the peroxisome proliferator-activated receptor gamma (PPARγ), our analyses suggest its potential as a novel biomarker and target for cancer prevention. Furthermore, we observed upregulated regulon activities for NCOR1, a frequently mutated gene, in stem-like cells of both cirrhosis and HCC (Fig. 3C), showing increased expressions along malignant progression (Fig. 3B). Additionally, GPX2, a glutathione peroxidase acknowledged for its upregulation in CRC^8^, exhibited elevated expression in HCC, ESCC, and cSCC, as well as their premalignant stages (Figure S2B). GPX2 functions to alleviate oxidative stress through the reduction of hydrogen peroxide, thereby facilitating both tumorigenesis and metastasis^13^.

To gain a comprehensive understanding of the TME landscapes, we next examined the cellular compositions and molecular features of immune, stromal, epithelial and endothelial lineages across different stage groups. Within the immune compartment (Figure S2C), we found that plasma cells (PLA), NK, and inflammatory monocytes (INMON) were highly enriched in precancerous lesions, and regulatory T cells (TREG), neutrophils (NEUT) and germinal center B cells (GC) in both precancer and cancer stage. M2 macrophages (M2MAC), CD4^+^ follicular helper (TFH), T-17 helper (TH17), and CD8^+^ terminally exhausted cells (CD8TEREX) were observed to be specifically enriched in cancers when compared to benign and healthy tissues (Fig. 3D). These implicated the potential mechanisms in facilitating immune evasion and inducing immune tolerance in more malignant tissues. SCENIC analysis revealed the higher activity of GATA3, IKZF2, BATF, RORA and FOXP3 in Tregs of each stage across tissue types (Table S3). In addition, HLF and ARID5B were identified as TFs of Tregs in precancer stages (e.g., N_HPV, AAH, NEOLP, EOLP), while regulons such as FOXO1 and STAT5B were specifically detected in cancers (e.g., IDC, AIS, MIAC, IAC and PDAC). Similarly, for INMON, FOXO3 and IRF5 were common TFs in healthy and early-stage tissues (e.g., BRCA1-mut); specifically, PRDM4 was the TF in precancer stages (e.g., Goiters and HT) while ATF2 and RUNX1 were enriched in cancers (e.g., ESCC and IDC, Figure S2D, Table S3). Multiple types of fibroblasts and endothelial cells were identified for the stromal compartment (Fig. 3D, Figure S2C). Among distinct subpopulations of fibroblasts, myofibroblasts (MYOFIB) were found to be enriched in both PME and TME (Fig. 3D), while hepatic or pancreatic stellate cells (HSC/PSC), intermediate CAFs (INCAF) and pericytes (PERI) with high expression of RGS5 were significantly enriched in malignant stages (Fig. 3D). Additionally, upregulated TF activities in MYOFIB/HSC were noted for PATZ1 in preneoplastic stages (e.g., Cirrhosis, AK, and CAG), while CEBPZ and SOX6 demonstrated elevated activities in malignant (e.g., cSCC and HCC) stages (Figure S2D, Table S3).

We further calculated the fractional contributions of each cell type to each sample as a function of position in the malignancy index. Some cell subpopulations showed strong correlations with disease progression along the index. In the epithelia, stem-like cell fractions in the samples gradually increased throughout the malignant transformation process. Conversely, the number of enterocytes decreases as adenomas transformed into carcinomas. In the secretory compartment, we observe a decreasing trend in fractions of pit mucous cells (PMC) and chief cells (CHIEF) with neoplastic transformation of the pre-cancerous gastric mucosa. In carcinomas, there was a general lack of differentiation toward the secretory lineage, resulting in the elimination of goblet cells (GOB, Fig. 3E). This aligns with a previous study documenting a reduction in goblet cells in non-mucinous colon adenocarcinomas^8^. Knocking down MUC2, a mucin protein associated with goblet cells, resulted in an increased formation of adenomas and carcinomas in mice^8^, implying that the loss of immature and mature goblet cells could potentially contribute to tumorigenesis^14^. In the immune cell compartment, the proportions of NK, dendritic cells (DC) and progenitor exhausted CD8 + T cells (CD8TEXP) in liver tissues decreased as the disease progressed. However, GC cells increased in the colorectum and esophagus, TREG in the breast and M2MAC in the pancreas increased (Fig. 3E, Figure S2E). To further evaluate the functional variations in these evolving microenvironment cells as disease escalated, we analyzed the curated gene signatures (See Methods) and mapped the related molecular changes along malignancy continuum. AUCell algorithm^11^ was designed to score the activity of a gene set in each cell. Here we utilized AUCell to delineate the transcriptional patterns of five major cell types. For instance, as the malignant progression occurred in liver, lung, oral cavity and thyroid tissues, CD8 + T-cell activation & effector molecules (e.g., FGFBP2, CX3CR1, FCGR3A and KLRG1), high cytotoxicity gene signature and T cell receptor (TCR) signaling gradually increased from the initial stages (Fig. 3G, Figure S2F). Moreover, we noted a progressively accelerated expression of stress response gene signatures (e.g., stress-related heat shock genes such as HSPA1A and HSPA1B), and the expression of nuclear factor (NF)-κB signaling, a key regulator of cellular stress response, was also enhanced (Fig. 3G). Advanced tumor tissues displayed increased expression of adhesion, IFN response, anergy and exhaustion-related signatures (Fig. 3G). In line with prior findings^15^, CD8 + TEX cells can express high levels of cytotoxicity markers, indicating likely antigen experience (Figure S2F, G). CD4 + T cells gradually expressed anti-apoptosis, effector function genes and TCR signaling signatures. They markedly expressed co-stimulatory molecules (e.g., TNFRSF4, TNFRSF9, TNFRSF18), adhesion, and IFN response signatures in the very late stage of cancer. The metabolic switch of T cells along disease progression was in accordance with their dynamic changes in activation and effector functions. Notably, CD4 + and CD8 + neoantigen-reactive tumor-infiltrating lymphocytes (TILs) exhibited distinct signatures across different stages (Figure S2G). In the examined tissues, advanced stages showed increased levels of neoantigen-specific T cell signatures along with higher mutational burden (Fig. 2F, Figure S1C, Figure S2F, G), indicating an active immune response against the tumor. T cell exhaustion, characterized by reduced cytokine production and increased expression of inhibitory receptors in response to chronic antigen stimulation (e.g., neoantigens, HBV, HPV and cancer testis antigens), has been recognized as a primary mechanism of immune escape by cancers^16,17^. Intriguingly, this trend of T lymphocytes was reversed during disease progression in the cervix, endometrium, pancreas, and prostate tissues (Fig. 3G, Figure S2F), with higher levels of NeoTCR8/4 signatures and mutation load found in the precancerous stage compared to those in advanced tumors (Figure S2G). We inferred that heterogeneous distribution and functional patterns of T cells during multistage tumorigenesis across various solid tissues mirror inter- and intra-tumor heterogeneity, emphasizing the broad impact of tissue types on the states of T cell subpopulations. Additionally, NK cells exhibited the lowest cytotoxicity but the highest stress score in advanced tumors. Other TME cells also underwent drastic reprogramming during malignant transition, with macrophages shifted from the M1 to M2 state and fibroblasts transitioned from an inflammatory CAF (iCAF) to a dominant myofibroblastic CAF (myCAF) phenotype (Fig. 3F). These observations suggested that the stromal cell remodeling and immune suppression in the TME accompanied the acquisition of stemness by tumor cells and coincided with plasticity onset and progression.

In summary, our observations concerning the enrichment of innate immune cells, such as INMON and NK in the early precancerous phases reflect the activation of innate immunity and its rapid onset in response to harmful stimuli such as microbial infection. Various leukocytes (e.g., macrophages, NK, and DC) bridge innate and specific immunity and hold a dominant position in immune surveillance and clearance of abnormal epithelial cells. If pro-inflammatory stimuli persist during the epithelial wound-healing process, chronic inflammation may ensue, leading to the persistence of inflammatory factors and tissue damage. Various phenotypically distinct immune/stromal subclusters may dynamically shift and closely interact with other PME/TME cells, playing a critical role in tumorigenesis and cancer evolution.

### 4. Heterogeneity of transcriptional programs in stem-like cells.

**Figure 4:**
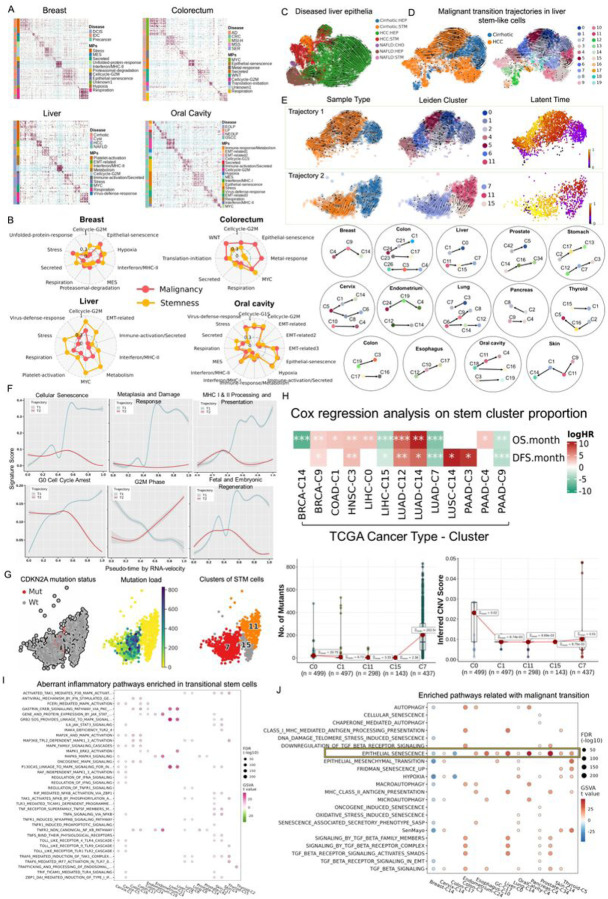
Transcriptional programs and precancer to cancer transformation routes. (A) Heatmaps of the significance of the overlap between individual tissue modules (hypergeometric test p-value) in four types of tissues. The bar indicates the annotation for disease states and modules. Programs are ordered by clustering and grouped into MPs. (B) Correlation of MPs with stemness and malignancy index. UMAP plots showing the inferred development dynamics of diseased liver cells by RNA velocity colored by sample type & cell type; (C) and of stem-like cells colored by sample type (D, left panel) and Leiden subclusters (D, right panel). (E) Top panel, RNA velocity indicating two trajectories of stem-like cells involved in cirrhotic to HCC transition. From left to right, they are colored by sample/cell type, Leiden clusters, and latent time. The bottom panel showing simplified trajectories across 13 tissues inferred by RNA velocity analysis. (F) Line plots of changes for the representative transcriptional programs over pseudotime. (G) Left panel, stem-like cells from trajectory 2 of liver tissues labeled with genomic alterations in CDKN2A (left), mutation load (middle), and Leiden clusters (right). Right panel, difference of genomic profiles (including mutations and copy number alterations) among five representative clusters in two trajectories of neoplastic transformation of cirrhosis. (H) Cox regression analysis showing that the OS and DFS in TCGA patients were significantly stratified by the median proportion of stem subclusters. (I) Aberrant inflammatory pathways enriched in transitional stem clusters at the start point in corresponding malignant transition trajectories. (J) Representative pathways enriched in malignant or benign stem clusters at the endpoint in related transition paths.

Cancer cells exhibit heterogeneity in their degree of differentiation, ranging from stem- or progenitor-like to fully differentiated. Gene expression programs are often used to characterize different cell identities and cell activities. To better understand advanced and highly heterogeneous tumors, we investigated the intratumor heterogeneity (ITH) programs through transcriptomic profiling of stem-like cells from tumors and their originating lesions. We then characterized the expression programs for each diseased sample using non-negative matrix factorization (NMF) approach (see Method details)^9,18^. Of note, the number of programs varies for each disease across the 13 tissues. To nd the recurrent patterns of ITH in stem-like cells and decipher consensus modules consisting of coexpressed genes within all expression programs, we next clustered these coexpressed genes to gene modules using K-means clustering (Fig. 4A, Figure S3A). After filtering out gene modules annotated with ‘unknown’ (due to low quality data or doublets), we summarized a total of 30 ITH programs across 13 tissues (Fig. 4A, Figure S3A), named as meta-programs (MPs). For each sample, we used AUCell score to represent the activity of each MP and then calculated the correlations between 30 expression programs and stemness (measured by CytoTRACE). The stronger correlations (R > = 0.7) were observed for MYC, Hypoxia, Cellcycle-G2M, Translation-initiation, EMT-related, Epithelial-senescence, Metabolism and Cellcycle-G0Arrest (Fig. 4B). More specifically, Cellcycle-G2M in the oral cavity and skin tissues, Epithelial-senescence (EpiSen) and Hypoxia in oral cavity, MYC in colorectum, Translation-initiation as well as Cellcycle-G0Arrest in thyroid were found to concurrently and highly correlated with our defined malignancy index and stemness (Fig. 4B, Figure S3B). This indicated that these six MPs were crucial for acquisition of stemness and tumorigenic properties during malignant transformation. We found that MPs may be expressed variably or uniformly in a given precancer/cancer sample across different tissue types, but the extent to which the expression of these critical programs varies along the process of tumorigenesis remains to be uncovered.

To better understand the mechanisms governing the generation of precancer cells as well as their malignant transformation, we sought to unravel the distinctive features acquired or lost during the progression from benign to malignant stages. We first utilized Leiden algorithm^19^ to cluster all diseased epithelial cells into multiple subclusters (e.g., 24 clusters identified in liver-diseased tissues, Figure S3C). Next, we employed RNA velocity analysis to delve into those subclusters of diseased epithelia and infer epithelial cell transition trajectories (Fig. 4C). However, when we combined data from multistage epithelial cells (e.g., HCCs, NAFLD, and cirrhosis), ITH increased significantly as liver epithelia transitioned from precancerous to cancerous states (as shown in Fig. 4C). It suggested that stem-like cells in HCC may arise from cirrhotic stem cells, subsequently differentiated into HCC hepatocytes. Malignant hepatocytes also appeared to acquire a degree of stemness and engaged in dedifferentiation into a stem cell-like state (Fig. 4C). We thus focused on the critical cell subpopulations (i.e., stem-like cells) involved in the malignant transformation of 13 diseased tissues. We turned to performing Leiden sub-clustering analysis using stem-cell expression data from precancer and cancer samples, thereby reducing the intrinsic complexity and heterogeneity of diseases by dissecting state transition in cancer cell-of-origin. The number of stem cell subpopulations identified in different tissues varied. For example, 20 stem-cell subclusters (ranging from C0 to C19) were identified in the liver (as shown in Fig. 4D). To gain further insights into the transition trajectories within these identified subclusters, we conducted a subsequent RNA velocity analysis to elucidate their directionality embedded on a diffusion map (Fig. 4D).

Similar analyses were carried out across 13 tissues, identifying one to three trajectories within each (Fig. 4E, Figure S3C). For instance, two pronounced directional flows were observed from cirrhotic stem-like cells (C1 and C11) towards HCC subclusters (C0 and C7), respectively (Fig. 4E). Along the C1 to C0 transition path (Trajectory 1, Fig. 4E), notable increases were observed in cellular senescence, damage response, MHC I & II processing and presentation, fetal signatures, and cell cycle G0 arrest signature scores (Fig. 4F). Interestingly, a notable uptick in mutational load within C0 compared with C1 was noted (Fig. 4G). This observation is consistent with that MHC-II^high^ stem cells represent a deeper quiescent state and are more resistant to stress-induced proliferation than MHC-II^low^ stem cells^20,21^. These findings underscore the pivotal role of cellular senescence in tumorigenesis and suggest that mutation-accumulated stem cells in C0 gained a clonal advantage during aging by upregulating their surface expression of MHC class II molecules. In addition, RNA velocity analysis showed cirrhotic stem cells in C11 exhibited another directional flow toward C15 and C7 (Fig. 4E). Along C11 to C7 (Trajectory 2), decreased cellular senescence, fetal and quiescence signature but increased S and G2M cell cycle scores were observed (Fig. 4F), indicating restoration of proliferative capacity. Escape from proliferation arrest had been reported in certain circumstances, such as reactivation of telomerase activity or deletion of CDKN2A^22^. Genomic events regarding C7 HCC cells harboring CDKN2A mutations and displaying the highest mutation load further supported the resumption of proliferation of stem-like cells in a quiescence-(or G0)-like state (Fig. 4F, G).

Next, we shifted our attention from stem-like cell clusters to clinical implications within them. Using our integrated scRNA-seq profile as a reference, we profiled the cellular components of each sample in the corresponding TCGA cohorts using CIBERSORTx^23^. The results showed that the cellular composition inferred by deconvolution correlated with clinical features of TCGA patients. Particularly, the percentage of several transitional populations showed significant difference in tumor and adjacent normal tissues and notable associations with clinical features, mutations, and TME components of TCGA tumors (Figure S3D, Table S4). These inferred stem-cell proportions could also predict the OS or DFS of TCGA patients, such as C9 and C14 in breast, C1 in colorectum, C0 and C15 in liver, etc. (Fig. 4H). Compared with healthy stem cells, several transitioning clusters from precancer tissues were enriched in signaling pathways that regulate inflammation initiation, such as NF-kB signaling, JAK-STAT, toll-like receptor (TLR) and mitogen-activated protein kinase (MAPK) pathways (Fig. 4I, Table S5). This implicated the crosstalk of precancer stem cells between tumorigenesis and inflammatory processes. Most intriguingly, EpiSen was inevitably enriched in critical transitional stem cell populations involved in malignant transformation across 13 tissues (Fig. 4J), which was reminiscent of senescence-associated stemness. In addition to EpiSen, many malignant stem-like cell clusters exhibited strong enrichment of EMT, Hypoxia, TGF-β pathway and Autophagy (e.g., C14 in the cervix, C0 in the liver, C14 in the lung, C4 in the pancreas and C5 in the thyroid, Table S5), indicating that hypoxia signaling appeared to involve in the activation of stem cell stress signaling by expressing some stemness regulation genes, such as TGF-β, to induce dormancy, stimulate autophagy, promote cell survival and maintenance of stem cell identity, as well as promote EMT^24^. The inferred fractions of these dormant stem cells were significantly correlated with higher TGF-β response in TCGA bulk data, further validating the observations from our single-cell analyses (Figure S3E). Nevertheless, the mere detection of a senescence-like cellular state, e.g., in BRCA1+/mut breast tissues (C14), colon adenomas (C17) and other malignancies, does not indicate whether these cells have a tumor-suppressive or pro-tumorigenic functional role.

### 5. Alterations in cell-cell interactions over time and master pro-tumorigenic mediators.

**Figure 5:**
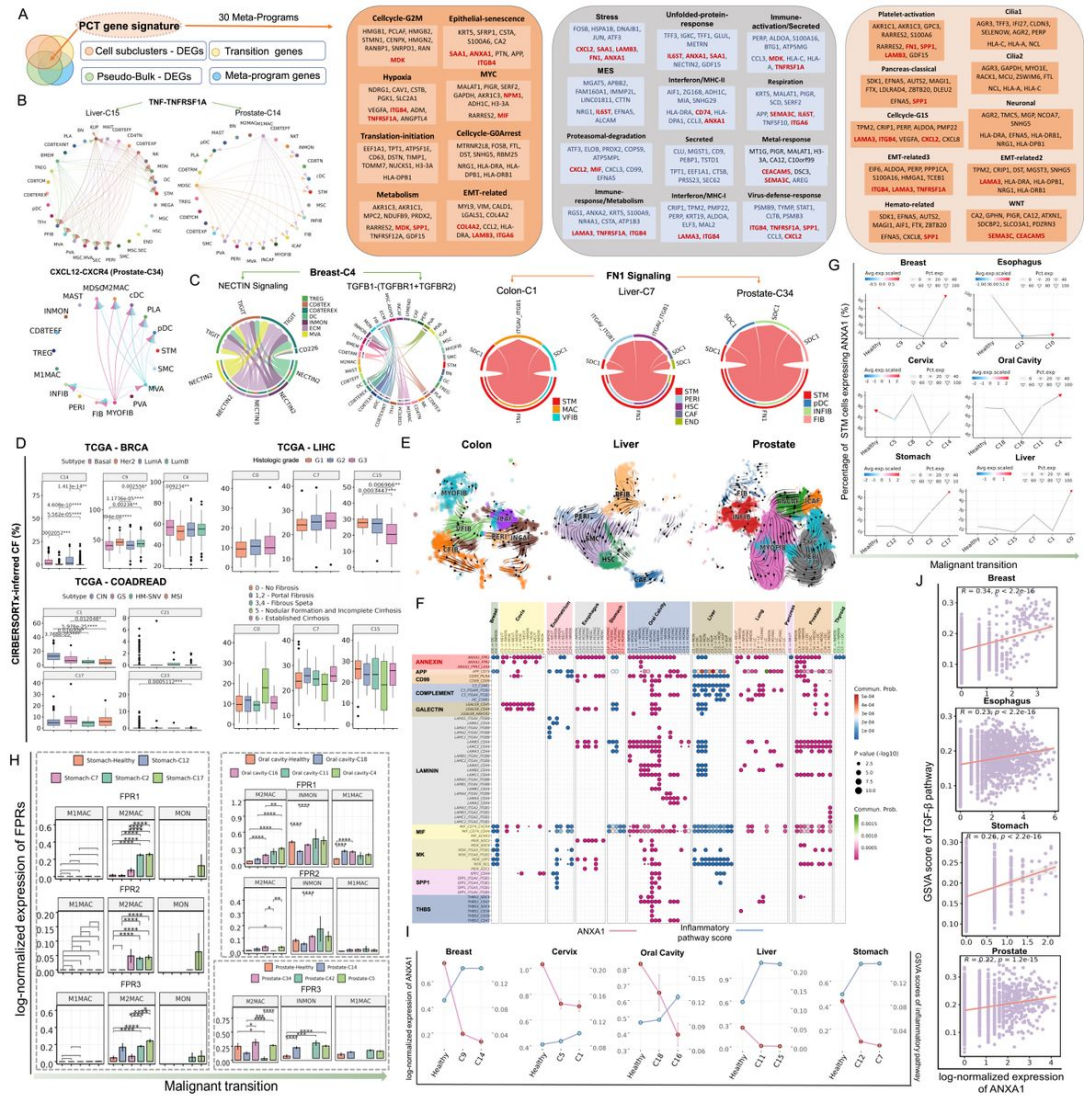
Alterations in cell-cell communication along pseudo-time axis and identification of master pro-tumorigenic mediators. (A) The left diagram shows the generation process of the PCT gene signature. Right, ten representative genes in 30 defined MPs. (B) Significant cell-cell interactions of TNF and CXCL pathways were identified in PME cells. (C) Cell-cell interactions of NECTIN and TGF-β (left) pathways identified in TME cells from the Breast C4 stage and of FN1 signaling identified in TME cells from malignant stages of colon, liver, and prostate tissues. (D) CIBERSORTx-inferred proportions of stem cell clusters varied among different subtypes of TCGA tumors. (E) RNA velocities overlaid on the UMAP of stromal cells from colon, liver, and prostate tissues, respectively. Arrows show the RNA velocity field. Dots are colored according to stromal cell subsets. (F) The dot plot showing representative ligand-receptor pairs significantly enriched in communications between transitioning stem-like cells and myeloid subsets. (G) The dot-line plot showing the dynamics of log-normalized ANXA1 RNA level in transitional stem cells along malignant transformation. (H) Bubble chart showing the gene expression levels of formyl-peptide receptors (FPRs) in the main cell types along malignant transitions based on the scRNA-seq data. (I) The dot-line plots showing the inflammatory pathway scores and the ANXA1 RNA levels in stem clusters from the start point of transition trajectories. (J) The Scatterplots for correlations of ANXA1 expression levels with the GSVA scores of TGF-β signaling.

To comprehensively compare and quantify the differences between cellular states in malignant transformation, we identified DEGs between each pair of core clusters from each trajectory, such as C4 v.s. C9, C9 v.s. C14 in breast, C0 v.s. C1 and C7 v.s C15 in the liver (avg_log2FC > = 0.25, pct.1 > = 0.25, and pct.ratio > = 1.5). In addition, we endeavored to detect the transition genes by calculating Spearman’s rank correlation between the inferred pseudo-time and gene expression of all stem-like cells along each trajectory (with FDR < 0.05). Subsequently, based on the consensus of related MPs, we generated gene signatures that specifically reflect characteristics of different stem cellular states. Spearman correlation analyses were performed on gene expression levels and AUCell scores of each MP. Genes significantly correlated with AUCell scores (FDR < 0.05) were considered meta-program genes. To obtain specific gene signatures involved in malignant transition, we identified (1) DEGs that distinguish the diseased stem-like cells from normal-like cells (Pseudo-Bulk – DEGs, FDR < 0.05 and log_2_FC > 0.25), (2) DEGs between stem-cell subcluster, (3) transition genes, and (4) MP genes, and then intersected them to generate malignant transformation-related (PCT) gene signatures (Fig. 5A). Subsequently, the geometric mean of the Spearman correlation coefficients for each gene from 13 tissues were calculated. Finally, 100 top-ranked genes sorted by geometric mean value were filtered into PCT genes for each MP (Fig. 5A).

To seek for gene(s) in 30 core sets of PCT signatures that may drive stem-cell inflammation and aging^6^ during loss of tissue homeostasis and precancer development, we then analyzed the dynamics of stem cell crosstalk with the microenvironment along our identified pseudotime axis. We identified 989 significant ligand-receptor pairs which revealed intercellular communication between 62 transitioning stem cell subpopulations and corresponding immune and stromal cells using CellChat^25^ (Table S6). Several predicted interactions related to cytokines/chemokines in inflammatory response were frequently observed in epithelial stem cells and other PME cells during the transition from tissue homeostasis to pathological lesions. For instance, we observed extensive crosstalk of TNF-TNFRSF1A, CCL2-ACKR1, CXCL2-ACKR1, and CXCL12-CXCR4 in Breast-C14, Cervix-C5, Liver-C15, and Prostate-C14, suggesting their role in proinflammation and leukocyte recruitment (Fig. 5B, Figure S4A). This contribution likely leads to macrophage M1 polarization while inhibiting the invasion and migration of damaged epithelial cells^26^. It’s worth noting that these ligand-receptor interactions involved in wound repair after tissue damage (like CXCL12-CXCR4 axis), potentially exert a two-edged effect in regulating anti-tumor immune responses (e.g., Prostate-C14 and Oral cavity-C18) and promoting tumor cell growth (e.g., Prostate-C34 and Liver-C0, Fig. 5B, Figure S4A)^27^. We also noticed unique cellular crosstalk at different stages. Strikingly, interactions between the TIGIT receptor in lymphocytes (e.g., TREG and CD8TEREX) and NECTIN2/3 ligands in dendritic, mesenchymal and endothelial cells were found at C4 of breast cancer, but not at C9 and C15 stages of preneoplastic BRCA1+/mut breast tissues, showing the multiple immunosuppressive signals in the Breast-C4 TME. Additionally, FIB, INMON, and DC expressed TGFB1 to communicate with ECM-receptor, indicating that TGF-β signaling of C4 tumors may regulate fibroblasts activity and modulate ECM production and components (Fig. 5C, Figure S4B). Furthermore, interaction between stem cells and ICAF, INCAF, VFIB, PERI, HSC, INFIB or END via FN1-ITGAV + ITGB1 and FN1-SDC1 were detected in Cervix-C8, Colon-C1, Liver-C7, Oral calvity-C3 and Prostate-C34 (Fig. 5C). This indicates that extensive stromal remodeling occurs during tumorigenesis in infected or injured tissues, acquiring EMT-like features (e.g., FN1) in transitioning stem-like cells and showing high expression levels of SDC1 in fibroblasts, which is linked to aggressive phenotypes and poor survival (Fig. 4I, Fig. 5D, Figure S4C). FN1 from the Stress and Platelet-activation program was highly expressed in those ‘hybrid’ cells undergoing plasticity changes, but somewhat less in other transitional stem clusters (Fig. 5A, Figure S4C). This finding suggests that during physiochemical stress situations, pro-tumorigenic fibroblasts in close proximity can provide the fertile ‘soil’ to the cancer ‘seed’, further influence platelet activation, which produced pro-angiogenic and growth factors that facilitate tumor growth and survival, as well as promoting the metastatic potential of tumor cells^28^. In addition, dynamic shift of HSC/VFIB to SMC/MYOFIB that highly expressed ACTA2 and MYH11 was observed in the liver, colon, and prostate diseased tissues (Fig. 5E), consistent with the aforementioned accumulation of MYOFIB and HSC in precancerous stage (Fig. 3E). These observations were also in line with that type I collagen, enriched in activated myofibroblastic HSC, promoted proliferation, tumor development and related to elevated HCC risk in patients^29^.

Notably, several ligand-receptor pairs such as LGALS9-CD44/CD45, laminins-integrins and MIF-CD74 + CXCR4 were significantly enriched in the communication between transitional stem cells and myeloid subsets (e.g., NEUT, INMON, M2MAC, etc., Fig. 5F). The MIF-CD74 + CXCR4 interaction can exert an immunosuppressive role by affecting downstream MAPK signaling pathway effectors^30^. Most interestingly, ANXA1-FPR1/2 of the ANNEXIN pathway was activated in the malignant transition of 11 tissues (Fig. 5F). ANXA1, one of the representative genes in four stem-cell MPs (i.e., EpiSen, Stress, Unfolded-protein-response and Interferon/MHC-II), is highly expressed in most healthy human tissues, but silenced in nonalcoholic steatohepatitis^31^ and early onset of esophageal and prostate carcinoma^32^. The expression levels of ANXA1 in stem cells decreased from the healthy to the precancerous stage but gradually increased along our identified malignant transformation paths (Fig. 5G, Figure S4D). The expression of ANXA1 receptor genes, including FPR1, FPR2, and FPR3, was enriched in myeloid cells and elevated in most malignant stages (e.g., M2MAC and INMON, Fig. 5H, Figure S4D). Additionally, we observed a pronounced elevation in the inflammatory response scores of transitioning stem subpopulations at the starting point of trajectories when ANXA1 expression was depressed during tissue damage (Fig. 5I). Conversely, as malignancy progression, positive correlations were noted between higher ANXA1 expression levels and the GSVA score of the TGF-β signaling, implying the involvement of ANXA1 in promoting TGF-β activation from epithelial stem cells (Fig. 5J, Figure S4E).

### 6. Spatiotemporal ANXA1 expression patterns in heterogeneous tumor ecosystems facilitate immunosuppression of the microenvironment.

**Figure 6:**
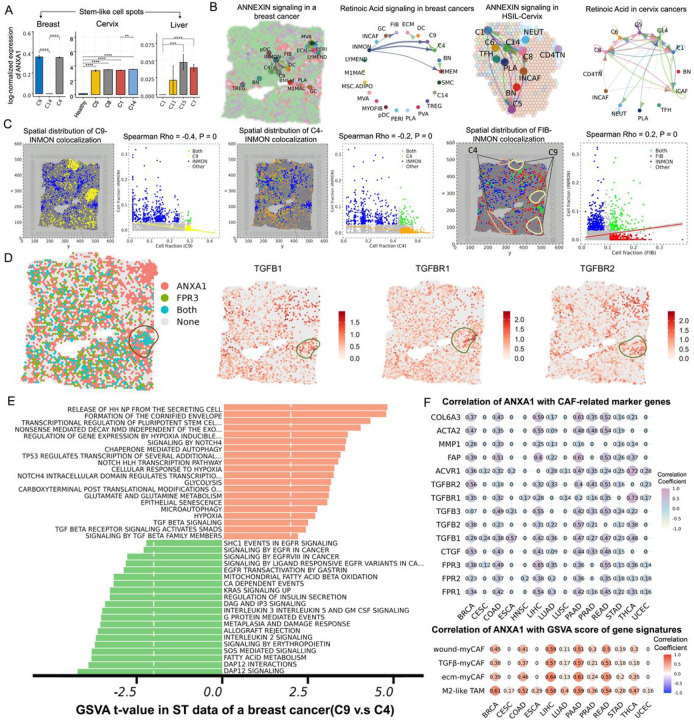
Spatially-resolved transcriptomics data revealing the transitional cell heterogeneity and potential mechanisms responsible for both mediation of stem cell senescence and immune suppression induction. (A) Barplots showing log-normalized ANXA1 RNA level in spatial stem-like spots of different stage samples from three tissue types. (B) Spatial plots presenting significant ANNEXIN and Retinoic Acid signaling networks identified in breast and cervix tissues. (C) Spatial distribution of C9-INMON (left), C4-INMON (middle) and FIB-INMON (right) colocalization in a breast cancer sample. Each dot represents an ST spot. (D) Visualize ANXA1-FPR3 and TGF-β expression distribution on the breast cancer tissue. (E) GSVA showing the significant differential pathways between C9 (close) and C4 (distant) spots of a breast cancer samples. (F) Correlation analysis on expression levels of ANXA1 with CAF-related makers (top) and the GSVA scores of four gene signatures (bottom) based on TCGA bulk data.

To further investigate the spatial architecture of transitioning stem subpopulations and distinct TME clusters during disease progression, we analyzed the spatial transcriptome (ST) data from breast, cervix and liver-diseased tissue samples. The cell components in each spot were determined by SpaCET using our integrated scRNA-seq data as the reference. We also deconvoluted the cell-type composition of each region in one HPV-negative normal cervix sample based on scRNA-seq data from the healthy cervix. The dynamic expression levels of ANXA1 were confirmed by ST data analysis, demonstrating a substantial increase in the bulk levels of ANXA1 RNA (located in both healthy epithelium and tumor spots) along malignant transition routes (Fig. 6A). Since ST data has not yet reached single-cell resolution, ANXA1 also exhibited strong expression in myeloid and mesenchymal spots of cancers and elevated in advanced stages (Figure S5A). It is still challenging to validate several transitional stem clusters with ST data (e.g., Liver-C0). Under these circumstances, tumor epithelial regions may have both stem-like and CAF features. As expected, activation of the ANNEXIN pathway and Retinoic Acid (RA) were observed among stem-like, myeloid, and mesenchymal subclusters, as inferred through spatially-proximal cell-cell communication analysis using CellChat V2 (Fig. 6B, Figure S5B). These findings are consistent with previous studies highlighting the role of CAF-secreted ANXA1 in imparting stem cell-like properties to cancer cells^33^. Additionally, research has shown that the N-terminal peptide of AnxA1 initiates a signaling cascade through FPR2, leading to the polarization of macrophages towards the M2 phenotype^34^. Subsequently, we aimed to delve into the spatial heterogeneity of tumors in more depth. Our results showed that the spots of FIB-INMON interactions with co-expression of ANXA1-FPR3 and TGFB1-TGFBR1/2 in the breast tumor sample are close to the C9 but distant to the C4 population (Fig. 6C, D, Figure S5C). Two different cancer cell states in ST data displayed distinct enrichment pathways, consistent with GSVA analysis of scRNA-seq data. Proximal C9 cells clustered separately from other tumor cells and showed senescent phenotype, while distal C4 spots exhibited hypermetabolic features and higher activity of oncogenic pathways, such as KRAS and EGFR signaling as well as DAP12 interactions (with myeloid cells) (Fig. 6E). We speculate that the senescent state of C9 cells in breast tumors is triggered by hypoxia and dysregulation of microenvironmental growth factors like TGF-β and NOTCH signaling. This, in turn, enhances ANXA1-FPR1/2/3 crosstalk, facilitating the reprogramming of fibroblasts and promoting the shift into an immunosuppressive environment from the PME. Last, we examined the bulk levels of ANXA1 RNA in TCGA data and observed a significant correlation of ANXA1 with CAF activation marker genes (FAP, ACTA2, COL6A3, MMP1 and TGF-β), wound healing-myCAF, ecm-myCAF, and TGFβ-myCAF gene signatures, as well as M2-like signatures of tumor-associated macrophages (TAMs) (Fig. 6F). This further confirmed that ANXA1 plays an important role in inducing immunosuppressive TME by mediating the transformation of normal fibroblasts into CAFs and macrophages M2 polarization. Additionally, our defined EpiSen signature was also significantly and positively associated with stromal cell fractions and TGF-β response in TCGA tumors (Figure S5D).

## Discussion

Malignant progression from precancerous stages to cancer, is driven by the sequential acquisition of genetic alterations in oncogenes and tumor suppressor genes, resulting in uncontrolled cell proliferation^35^. The “bad luck” theory posits that random mutations in self-renewing stem cells contribute to cancer development by generating malignant, self-renewing daughters that propagate cancer^36^. Emerging evidence underscores the role of extrinsic factors like inflammation, metabolism, and wounding in predisposing tissues to heightened cancer vulnerabilities^37^. However, the mechanisms through which oncogenic mutations in healthy tissue stem cells might trigger intrinsic environmental changes, potentially circumventing the need for multi-step mutagenesis, remain less understood.

In this study, we observed a progressive enrichment of stem-like cells within the epithelium as the lesion aggravated. The inherent “self-renewal” properties of these cells enable the maintenance of a stem-cell pool, indicating that they are long-lived and capable of dividing without differentiation^6^. Consequently, these specialized niches emerge as probable cells of origin for cancers and crucial regulators of cancer risk. Indeed, Chen et.al. proposed a perspective about adenomatous tumorigenesis in which neoplastic cells of colon tissues originate from DNA replication-induced mutations in continuously renewing stem cells^14^. In our single-cell analysis of 13 tissues, we compared the gene expression profiles of stem-like cells in diseased tissues with those in healthy donors. We proposed that these stem cells form a potential malignancy continuum; therefore, we estimated the pseudo-time along disease progression (ranging from healthy to cancer state) as the malignancy index. We believe that the changes in gene expression along this malignancy progression can implicate tumorigenesis mechanisms and help identify potential diagnostic and therapeutic targets for both dire precancerous lesions and malignancies. Notably, we observed that the frequently mutated gene NCOR1 exhibited upregulated regulon activities in stem-like cells from both cirrhosis and liver cancer. NCOR1 facilitates the interaction of several nuclear proteins that regulate the transcription rates of metabolic stress-induced genes^38^. NCOR1 functions as a negative modulator for hepatic de novo fatty acid synthesis (FAS) and mitochondria energy adaptation, playing distinct roles in regeneration or carcinogenesis^39^. Lee et al. found that chaperone-mediated autophagy dysregulation during liver aging impairs hepatic fatty acid oxidation through the accumulation of NCoR1^40^. Furthermore, we identified phenotypically distinct immune and stromal subclusters by delineating the composition and molecular changes of PME and TME cells along malignant transition. The dynamics of these subpopulations exhibited close associations with stem-like cells, potentially exerting influence on immune activation, homeostasis imbalance, exhaustion, and suppression over time and thus playing a crucial role in tumorigenesis and cancer progression.

Subsequently, we annotated the different stem cell states that recurred across tissue types using 30 transcriptional programs. These programs reflected current events that shape cellular states, such as cell cycle phases, and their short-term responses to surrounding cells, cytokines, and nutrients (or lack thereof)^9^. Thirty MPs also revealed a high degree of plasticity in epithelial stem cells. Among them, MYC, Cellcycle-G2M, EpiSen, EMT-related, and hypoxia strongly correlated with both malignancy and stemness. This suggests that some subpopulations may warrant innovative therapeutic approaches. For instance, combination therapies may target coexisting cellular states, while differentiation therapies may potentially transition cells from an aggressive state (e.g., massively proliferative and invasive) to a more benign or responsive state (e.g., senescent cells hypersensitized to microenvironmental IFNγ)^41^. Our velocity analysis unveiled that within malignant transition trajectories, multiple populations of senescent stem cells exhibited concurrent activation of hypoxia signaling and TGF-β, creating an oncogenic milieu with significant pro-tumorigenic effects. It has been proposed that TGFβ can stabilize HIFα protein; under chemotherapy-induced stress, HIF1α can enhance glutathione synthesis, promote the acquisition of CSC phenotype, and reshape the CSC population in tumors by constraining their differentiation capacity^42^. We argue that those identified senescent clusters with poor prognosis appear to be early-stage malignant progenitors that entered into proliferative quiescence in response to TGF-β. Therefore, TGF-β-induced dormancy might protect stem-like metastasis-initiating cells from immune surveillance, preserving these cells for eventual relapse^43^.

We observed that ANXA1 is highly expressed in healthy humans and that the dynamic expression level of ANXA1 is closely related to malignant transformation. Increasing evidence indicates that AnxA1 triggers macrophage reprogramming towards a resolving phenotype, serving as a pivotal step in restoring tissue homeostasis^44^. AnxA1 functions by inducing neutrophil apoptosis, modulating monocyte recruitment, and enhancing the macrophage clearance of apoptotic cells. However, the expression of ANXA1 was reduced in epithelial stem subclusters within the injured tissues, and subsequently increased in malignant stem cells that exhibit excessive senescence or proliferation. This suggests a unique role of ANXA1 in precancerous stages, where its reduction contributes to increased production of proinflammatory cytokines, leading to aggressive and/or prolonged inflammatory responses^45^. In contrast, in carcinomas, ANXA1 expression gradually increases as lesions approach malignant transformation to allow and promote the transformation of fibroblasts into CAFs (enhanced by increased TGF-β production), thereby driving malignancy possibly through FPR activation^46^. This aligns with prior research demonstrating that AnxA1 deficiency exacerbates insulin resistance and metabolic impairment in mice on an obesogenic diet^47^ and aggravates lobular inflammation and hepatic fibrosis in experimental NASH^48^. These effects are associated with enhanced macrophage recruitment as well as their pro-inflammatory M1 phenotype and activity^48^. Furthermore, both intracellular ANXA1 and externalized ANXA1 were reported to be involved in tumor growth and involved in invasion process^46^. Our spatiotemporal analysis suggested that immunosuppression in the TME promoted by ANXA1/FPR interaction could be a strategy for tumorigenesis and progression. In this context, different cell subpopulations may dynamically cooperate within the tumor ecosystem, leading to higher fitness of the tumor as a whole. Specifically, under the downregulated states of ANXA1 expression in the PME, precancer stem cells acquire resistance to apoptosis and programmed cell death, thereby ensuring their longevity, and evading innate and adaptive immune responses. This leads to the generation of self-renewing CSCs, in part by becoming dormant in protective microenvironments. These cells can further employ an active self-protective mechanism by entrapping local myeloid cells and T cells to construct a prosurvival niche, obtaining a clonal advantage. For example, within TME, increased activation of retinoic acid (RA) can drive intra-tumoral monocytes differentiated toward TAMs but shift away from differentiating into DCs via suppression of IRF4^49^; ANXA1 promotes alternative macrophage polarization to enhance breast cancer growth^50^. Macrophages compete with DCs to degrade the tumor-associated antigens (TAA), preventing the initiation of antigen presentation and inducing immune tolerance^51^. Moreover, AnxA1, known to regulate the nuclear localization of EGFR, promotes T cell dysfunction by favoring the EGFR/STAT3 transcriptional activities in cancer cells, enhancing immune evasion and tumor progression^52^. Our analysis showed that fibroblasts activated in response to inflammation in the PME would persist and transform into CAFs characterized by high expression of ANXA1 levels and TGFβ-myCAF signatures in the TME. This indicated that quiescent CSCs might inhibit DC infiltration and promote Tregs expansion and the functional reprogramming of TAMs and CAFs, further contributing to the exhaustion of CD8 + T cells. Hence, through these mechanisms, senescent cells (e.g., Breast-C9) may mediate the downregulation of immune surveillance to benefit other proliferative cells (e.g., Breast-C4). However, apart from related mediators such as ANNEXIN and RA signaling, the mechanisms underlying TGF-β activation and the cooperative effects of minority senescent cells in promoting population-wide cancer cell survival remain to be determined.

**Figure 7:**
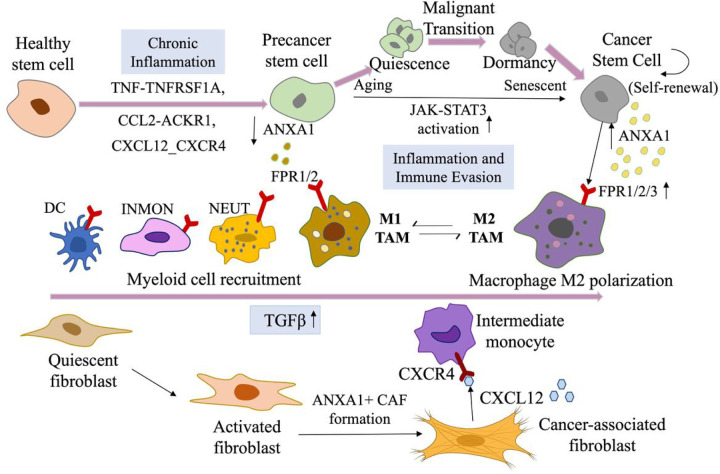
Schematic diagram to conclude our results. Due to the loss of ANXA1, inflammatory cells (e.g., monocytes) were recruited to the precancer stem-cell pool via aberrant inflammatory pathways, including NF-κB, MAPK, and JAK-STAT. During the progression from precancer stem cells to cancer stem-cell propagation, the expression level of ANXA1 was gradually increased; macrophages with increased expression of FPR1/2 (receptor of ANXA1) are polarized into the M2 phenotype; quiescent fibroblasts in the normal tissues are transitioned into activated fibroblasts, and then transitioned to CAFs in response to TGF-β. All these changes lead to immune evasive dormancy as well as further self-renewal of malignant stem cells and leading to a more immunosuppressive microenvironment.

In conclusion, by analyzing data from 1396 samples across 13 major tissues, we dissected the heterogeneous microenvironment during malignant transformation. We identified 30 recurring cellular states strongly associated with malignancy in transitional stem cell-like subpopulations highly enriched in precancerous and cancer stages. By analyzing spatiotemporal ANXA1 expression patterns, we further confirmed the potential mechanism of ANXA1 in manipulating tissue microenvironments by mediating immune response through recruitment of the neutrophils and monocytes, which skew to M1 macrophages in the PME, and by promoting immune suppression that favors M2 macrophage polarization and transformation of normal fibroblasts into CAFs during malignant development (Fig. 7). Our results provided a systematic view of cancer origins, and suggested that restoring and maintaining the balance of inflammation and their mediators (e.g., AnxA1/FPRs signaling) may represent a novel approach to control the evolution of precancerous lesions and mitigate the risk for cancer development.

## Limitations of the study

First, the lack of matched data between precancerous and cancer samples across the 13 tissues hampers single-cell lineage-tracing analysis of genetic subclones, as it relies on inferred CNV and mutations from the same patient. Second, despite a sufficient sample size for analyzing tumor initiation mechanisms after excluding the metastases, potential prior treatments in included cancer specimens may influence the observed tumorigenesis landscape. Third, intra-patient heterogeneity, particularly a modest number of epithelial stem cells in specific tissues, presents challenges for malignancy transformation analysis. Fourth, our analysis primarily concentrates on the precancer-to-cancer transition, leaving aspects like local expansion, metastasis, and therapeutic resistance to be explored. Hence, further investigations in more refined patient, animal and organoid data are essential for a comprehensive understanding of dynamic transition in TME cells and their remodeling in various contexts. Lastly, the absence of experimental validations may limit our ability to fully support multiple observations described in the manuscript.

## Methods

### 1. Data curation, Preprocessing, and Quality Control.

A total of 62 scRNA-seq datasets, which include 1396 samples (healthy, adjacent non-cancer, premalignant lesion, and cancer specimens) were curated to build a tumorigenesis atlas. These datasets were obtained from previously published studies, covering 13 major tissue types (that is, breast, cervix, colorectum, endometrium, esophagus, liver, lung, oral cavity, pancreas, prostate, skin, stomach, and thyroid)^29,53–59^. We excluded the distant metastatic tumor samples (e.g., colorectal liver metastases) for lineage-specific cell clustering. The data accession numbers and references for these datasets are provided on the website (https://ccsm.uth.edu/PCTfuncDB/). Before conducting downstream analysis, we excluded cells with low-quality transcriptomes following similar filtering steps. Specifically, cells with detected genes < 200 and > 7,000, and cells with more than 25% mitochondrial reads were removed. The doubletFinder_v3() function from DoubletFinder (version 2.0.3) was used for each sample with principal components set to be 1:20, nExp was set to 0.08 × nCells2/10,000, pN to 0.25 and pK to 0.09. We applied two commonly used batch correction algorithms, harmony (version 0.1.1) and BBKNN (version 1.5.1) to evaluate the significance of the batch effect. After rigorous doublets and batch effects removal, the integrated single-cell transcriptomes for three major cell types (epithelial, immune, and stromal) were retained, respectively.

### 2. Construction of Healthy Epithelial Reference and Projection of Diseased Cells.

We first constructed a normal epithelial reference using epithelial cells from healthy donors. After normalizing the data with normalizeData() function in Seurat (version 4.3.0), the iterative LSI dimensionality reduction was computed using four total iterations (clustering resolution of 0.1, 0.2, 0.4, 0.8) based on a pipeline from Granja et al 2019^7^. For each iteration, the ribosomal protein genes, mitochondrial-encoded genes, and HLA genes were filtered out; the top 3,200 most variable genes were identified from the remaining genes. We computed term frequency-inverse document frequency (TF-IDF) transformation on these genes, performed SVD on the transformed matrix, and provided dimensions 1:32 of this reduction as input to Seurat’s shared nearest neighbor clustering a resolution of 0.1. Then, cell clusters were identified with an increased resolution for the next three iterations. Also, we computed the log(counts per million) transformation using the “edgeR” package (version 3.36.0) and found the top 3,200 variable genes across the clusters. A TF-IDF transformation was computed on these variable genes, and an SVD was then performed on the transformed matrix. Dimensions 1:32 were retained, and clusters were identified using functions findNeighbors() and findCluster() in Seurat. After the final dimensionality reduction, we found that using the iterative LSI approach with 32 dimensions allowed us to denoise the data and limit batch effect, which was useful for the projections. Similarly, as described by Becker et al. 2022^8^, this final LSI dimensionality reduction was provided as input to compute a UMAP representation of the data, and the cells were clustered using a resolution of 1.0. The resulting clusters were then annotated based on maker genes. The projection of cells into the LSI subspace defined for healthy liver epithelial cells was done following the procedure described previously. Briefly, when computing the TF-IDF transformation on healthy reference epithelial cells, we stored the colSums, rowSums, and SVD. To project cells from additional samples into this subspace, we first zero out rows based on the initial TF-IDF rowSums. We next calculated the term frequency by dividing it by the column sums and computed the inverse document frequency from the previous TF-IDF transformation. These were then used to compute the new TF-IDF. The resulting TF-IDF matrix was projected into the previously defined SVD. Cells were classified by identifying their 25 nearest neighbors in the LSI subspace using get.knnx in R and then classifying the cell as the most common annotation for those 25 nearest neighbors.

### 3. Expression Dynamics Analysis and Definition of Malignancy Index.

To delineate transcriptomic changes occuring on the phenotypic continuum from healthy tissues to precancer and to cancer, we performed expression dynamics analysis by identifying differentially expressed genes (DEGs) between stem-like cells in each pre-malignant sample and tumor sample. Then, we defined the malignancy index through principal component analysis on log_2_FC of DEGs between stem cells from all precancerous lesions and cancers against all healthy samples. This analysis is based on the long-held view of oncogenesis that cancer cells arise from an aberrant dedifferentiated stem-like state. DEGs displaying a Wilcoxon False Discovery Rate (FDR) ≤ 0.05 and |log2FC | ≥ 0.5 in ≥ 2 samples were considered significantly differential and retained for further analysis.

### 4. Non-negative matrix factorization (NMF) and module detection.

By using the NMF R package (version 0.23.0)^60^, the NMF algorithm was performed separately on the identified stem cells of each diseased tissue. All these steps were performed like previous studies^9,18^. Specifically, NMF was applied on each SCTransform result for different rank values, and then we extracted consensus modules for each optimal NMF rank that was identified for each sample. We utilized NMF programs in each sample to characterize the ITH patterns that vary among its stem-like cells, each summarized by its top-scoring genes.

### 5. Regulon network prediction.

The activity of transcription factor regulons was evaluated by SCENIC (version 1.0.1)^11^. In brief, regulons were detected by calculating the co-expression of TFs and genes, followed by motif analysis. AUCell score, ranging from 0 to 1, was then calculated by the algorithm for each cell to evaluate the activity level for each TF regulon. The regulon analyses were implemented independently for different main cell types.

### 6. RNA velocity and pseudotime analysis.

Individual sample BAM files were used to recount the spliced reads and unspliced reads using the “run-dropest” or “run10x” command in velocto (version 0.17.17). Then, the dynamic velocity model from scvelo (version 0.1.25)^61^ was used for the RNA velocity analysis. In brief, after the gene selection and normalization, the first- and second-order moments were calculated with scv.pp.moments() function. The full splicing kinetics were recovered with scv.tl.recover_dynamics() function and the velocities were obtained with scv.tl.velocity() function in dynamical mode. The velocities were projected onto diffusion maps and visualized as streamlines with scv.pl.velocity_embedding_stream() function. The spliced vs. unspliced phase portraits of individual genes were visualized with scv.pl.velocity(). The latent time of cells was obtained with scv.tl.recover_latent_time() function. For samples without BAM files, we ran monocle3 analysis by processing the raw scRNA-Seq UMI count matrix with R package Seurat (version 4.3.0). Raw UMI counts were normalized to total UMI counts per cell using the negative binomial regression method, and the top 3,000 highly variable genes were selected using the variance-stabilizing transformation (VST) method implemented in the “SCTransform” function^62^ in Seurat. Batch effects were corrected across samples using Harmony. We then used the “cluster_cells, learn_graph, and order_cells” functions with default parameters in the monocle3 R package (version 1.0.0)^63^. The pseudo-times were inferred by using pseudotime() function on the final monocle object.

### 7. Differentiation potential prediction with CytoTRACE.

The CytoTRACE algorithm developed by Gulati et al.^64^ is a scoring method inferring the relative developmental potential of single cells based on gene counts per cell. To obtain a score that represents its stemness within the given dataset, we performed CytoTRACE based on the default recommended settings with all epithelial single-cell transcriptomes of 13 tissues, including healthy, precancerous, and cancer samples.

### 8. Single-cell somatic mutation detection and Copy number variation (CNV) analysis.

SComatic is a tool that provides functionalities to detect somatic single-nucleotide mutations in high-throughput single-cell genomics and transcriptomics data sets, such as single-cell RNA-seq. We run SComatic to detect somatic mutations in scRNA-seq data with aligned sequencing reads in BAM format for all epithelial cells. The input BAM file contains the cell type barcode information in the cell barcode tag “CB” generated from Cell Ranger or other drop-seq tools. We also applied a fathmm filter^65^ to all cells. Based on this machine learning approach, each mutant of a single cell was assigned a score about the likelihood of a given SNV/INDEL to be pathogenic. Only variants computationally predicted to be pathogenic (fathmm score > 0.7) were retained in further analysis.

By using healthy epithelial cells as the reference data, the initial CNV value of each diseased cell was estimated by the infercnvpy method (version 0.4.2) based on the transcriptomic profiles as described by Puram et al.^66^. To evaluate the CNV level of each single cell to predict the malignancy of cells, we defined the CNV score by calculating the mean squares of CNV values across the genome.

### 9. Gene sets for functional enrichment analyses.

We primarily used signatures from MsigDB, including the following collections of gene sets: Gene Ontology (C5.GOBP, C5.GOCC and C5.GOMF), Hallmark (H) and REACTOME (C2.CP.REACTOME). We also added additional signatures (Metaplasia, Senescence and CancerG0Arrest) of epithelial cells curated from the literature^9,14,67^. Based on these GO terms and signatures, functional annotation on gene modules identified by NMF was implemented in the ‘clusterProfiler’ package. Additionally, five curated gene signatures of T-cell, myeloid cells, fibroblasts, and NK cells^68–72^ together with above-mentioned gene sets and our defined MPs were utilized to assess the signature scores in related cell subsets based on the AUCell, GSVA and ssGSEA method. Signatures with an FDR-adjusted P < 0.05 were considered significantly enriched. For pathway activity analysis on TCGA bulk data, GSVA was also used to evaluate the enrichment of related gene sets.

### 10. Cell-cell communication analysis.

We inferred cell-cell interactions (CCI) and constructed communication networks among our identified malignant-transitioning stem-like cells and other PME/TME cells using CellChatDB.human in CellChat (version 2)^25^. Then, we used the netVisual_circle() function to show the strength or weakness of CCI networks from the target cell cluster to different cell clusters. Finally, the netVisual_bubble() function shows the bubble plots of significant ligand-receptor interactions between the target cell cluster and other subclusters.

### 11. Spatial transcriptome (ST) data analysis.

We collected ST data from 4 cases of breast cancer, 1 case of the normal cervix, 1 HSIL_HPV, 3 samples of cervix cancer and 1 liver cancer. The SpaCET R package^73^ was used to perform deconvolution of spatial transcriptomic spots into cell types. The integrated single-cell data for each tissue type was used as the reference, while the spatial transcriptomics data was submitted as a query dataset. For spot-level cell-type assignment, spots were annotated for cell type according to the largest deconvolution proportion as inferred by SpaCET. In addition, we also used CellChat v2 to infer and visualize the potential CCI in ST data based on cell-type deconvolution results.

### 12. Statistical analysis.

Standard tests employed in the present study comprised Student’s t-test, Wilcoxon rank-sum test, Kruskal-Wallis test, and Chi-square test. These tests were utilized to assess variations in continuous target or categorical variables across different subgroups for comparison. The descriptions of statistical details and methods are indicated in the figure legends, text, or methods. P-values were computed with a two-sided and unpaired Wilcoxon rank-sum test. Routine statistical analyses of this study were performed in R v4.1.0, and a two-sided p-value below 0.05 was deemed statistically significant.

## Data Availability

All datasets analyzed in this study were published previously and publicly available. This work relied on curation and integrative analysis of external studies. Thus, the curated data from 57 studies are available at https://ccsm.uth.edu/PCTfuncDB/index.html, including accession numbers for the primary datasets and results of multiple downstream analyses. Additional datasets from unpublished studies will be added when possible. This paper does not report the original code. Any additional information required to re-analyze the data reported in this paper is available from the lead contact upon request.
